# Broad-Spectrum Anti-Adhesive Coating Based on an Extracellular Polymer from a Marine Cyanobacterium

**DOI:** 10.3390/md17040243

**Published:** 2019-04-24

**Authors:** Bruna Costa, Rita Mota, Paula Parreira, Paula Tamagnini, M. Cristina L. Martins, Fabíola Costa

**Affiliations:** 1 i3S–Instituto de Investigação e Inovação em Saúde, Universidade do Porto, Rua Alfredo Allen, 208, 4200-135 Porto, Portugal; bruna.costa@i3s.up.pt (B.C.); rita.mota@ibmc.up.pt (R.M.); parreira@i3s.up.pt (P.P.); pmtamagn@ibmc.up.pt (P.T.); cmartins@ineb.up.pt (M.C.L.M.); 2 INEB–Instituto de Engenharia Biomédica, Universidade do Porto, Rua Alfredo Allen, 208, 4200-135 Porto, Portugal; 3 IBMC–Instituto de Biologia Celular e Molecular, Universidade do Porto, Rua Alfredo Allen, 208, 4200-135 Porto, Portugal; 4 Faculdade de Ciências, Departamento de Biologia, Universidade do Porto, Rua do Campo Alegre, Edifício FC4, 4169-007 Porto, Portugal; 5 ICBAS–Instituto de Ciências Biomédicas Abel Salazar, Universidade do Porto, Rua Jorge de Viterbo Ferreira 228, 4050-313 Porto, Portugal

**Keywords:** cyanobacteria, extracellular polymer, released polysaccharides (RPS), anti-adhesive coating, surface modification, medical device associated-infections

## Abstract

Medical device-associated infections are a major health threat, representing about half of all hospital-acquired infections. Current strategies to prevent this problem based on device coatings with antimicrobial compounds (antibiotics or antiseptics) have proven to be insufficient, often toxic, and even promoting bacterial resistance. Herein, we report the development of an infection-preventive coating (CyanoCoating) produced with an extracellular polymer released by the marine cyanobacterium *Cyanothece* sp. CCY 0110. CyanoCoating was prepared by spin-coating and its bacterial anti-adhesive efficiency was evaluated against relevant etiological agents (*Staphylococcus aureus*, *S. epidermidis*, *Pseudomonas aeruginosa* and *Escherichia coli*) and platelets, both in the presence or absence of human plasma proteins. CyanoCoating cytotoxicity was assessed using the L929 fibroblasts cell line. CyanoCoating exhibited a smooth topography, low thickness and high hydrophilic properties with mild negative charge. The non-cytotoxic CyanoCoating prevented adhesion of all the bacteria tested (≤80%) and platelets (<87%), without inducing platelet activation (even in the presence of plasma proteins). The significant reduction in protein adsorption (<77%) confirmed its anti-adhesive properties. The development of this anti-adhesive coating is an important step towards the establishment of a new technological platform capable of preventing medical device-associated infections, without inducing thrombus formation in blood-contacting applications.

## 1. Introduction

Hospital-acquired infections (HAIs) are considered a major challenge in healthcare units worldwide, resulting in increased morbidity, mortality and medical costs. In the United States only, about 1.7 million cases of HAIs are reported annually, and more than half are associated with bacterial colonization and biofilm formation on medical implants [1]. The most prevalent microorganisms in these biofilms are the Gram-positive bacteria *Staphylococcus aureus*, *S. epidermidis* and *Enterococcus* spp., the Gram-negative *Pseudomonas aeruginosa* and *Escherichia coli*, along with the fungi *Candida* spp. [2,3]. Current treatments are based in prolonged systemic antibiotherapy, but have proven to be insufficient, often toxic, and can even promote bacterial resistance. In addition, these treatments are ineffective in preventing the need to remove the device [4]. The development of new materials and/or surface coatings that avoid bacterial colonization, and thus biofilm formation, is crucial to minimize this worldwide problem. 

In recent years, there has been growing interest in polymers from marine organisms that emerge as valid alternatives for a variety of medical uses [5,6,7,8,9]. There are, for example, a substantial number of reports on the use of these polymers as antimicrobial agents and/or as a basis for antimicrobial agents (chitosan [10,11,12] and others from algal origin [13,14]) or as anti-adhesives (Funoran from seaweed *Gloiopeltis furcate* [15], and polysaccharides from the green alga *Chlorella* [8] and from the cyanobacterium *Spirulina* [8]). In this context, cyanobacterial extracellular polymeric substances (EPS), mainly composed of heteropolysaccharides, have particular features that make them particularly suitable for such applications. These polymers may contain up to 15 different monomers, have a strong anionic nature due to the presence of uronic acids and sulphate groups, and exhibit high hydrophobicity conferred by the presence of ester-linked acetyl groups, peptidic moieties and deoxysugars [16,17]. Previously, we extensively characterized the extracellular polymer produced by a marine unicellular strain, *Cyanothece* sp. CCY 0110 (hereafter *Cyanothece*). This cyanobacterium is a strong EPS producer, releasing the majority of the polymer to the culture medium (as RPS—released polysaccharides) [18]. The *Cyanothece* polymer is composed by nine different monosaccharides, including the acidic hexoses galacturonic and glucuronic acid [18], has a sulphate content of about 11% and a peptidic content around 4% [19]. This amorphous polymer has a high molecular weight, is remarkably thermostable, and soluble in water [18,19], and was previously tested for controlled drug delivery [6,7]. The results obtained showed that *Cyanothece* RPS is a good candidate for the delivery of functional proteins (such as lysozyme) and can be used in combination with Arabic gum for the microencapsulation of vitamin B12 [6,7]. 

The present study aimed at developing a *Cyanothece’s* RPS-based coating (CyanoCoating) meant to prevent medical device-associated infections. CyanoCoating was challenged with relevant etiological agents and exhibited a broad-spectrum activity preventing the adhesion of all the bacteria tested, even in the presence of plasma proteins. Our findings also demonstrated that this coating is biocompatible and blood-compatible, being therefore suitable to be applied to a wide-range of surfaces.

## 2. Results

### 2.1. Biopolymer Production

In order to obtain the extracellular polymer that was subsequently used for the development of the coating (hereafter CyanoCoating), the cyanobacterium *Cyanothece* sp. CCY 0110 (hereafter *Cyanothece*) was grown in conditions previously described as promoting cell growth and consequently the amount of polymer released [18]. The amount of polymer obtained from *Cyanothece* grown in 2 L bioreactors was 1.8 ± 0.2 g per g of dry weight. 

### 2.2. CyanoCoating Production and Characterization

For the proof of concept of CyanoCoating, gold substrates (Au) were used due to its suitability for surface characterization techniques [20]. CyanoCoating, and a coating using the reference material, medical grade polyurethane (PU), were covalently bound through a polydopamine (pDA) layer that was directly polymerized onto the Au substrates. Under mild alkaline aqueous conditions, and in the presence of oxygen, dopamine polymerizes rapidly forming a pDA layer, whose catechol groups allows pDA linkage to the substrate [21]. In addition, the pDA supports a variety of reactions with other organic species allowing the linkage of additional layers, creating coatings [22]. PUs are one of the most commonly used biomaterials for medical devices production, namely for blood-contacting devices [23]. The advantages of PUs are their durability, elasticity, resistance to fatigue and even compliance, besides acceptance and tolerance by the human body [23]. 

Both coatings (CyanoCoating and PU) were applied by spin-coating. CyanoCoating, PU, and the linker layer of pDA alone were characterized in term of thickness (ellipsometry), wettability (water contact angle measurements), surface functional groups (infrared reflection absorption spectroscopy), topography (scanning electron microscopy) and surface charge (electrokinetic analyser).

#### 2.2.1. Thickness

**Figure 1:**
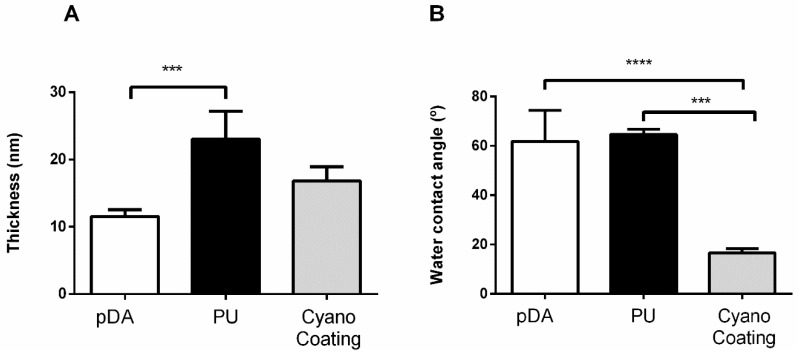
Surface characterization of Au substrates coated with a polydopamine (pDA) layer, a pDA layer plus polyurethane (PU), and a pDA layer plus the CyanoCoating by (**A**) ellipsometry (n = 9) and (**B**) water contact angle (captive bubble method) (n = 9). Statistical analysis was performed by non-parametric Kruskal–Wallis analysis and statistic differences are indicated with *** (*p* < 0.005) and **** (*p* < 0.001).

The CyanoCoating plus the pDA layer exhibit a 17 ± 2 nm thickness (Figure 1A). Since the samples with the pDA layer only presented a thickness of 11 ± 1 nm (Figure 1A), as a result of 2 h immersion in dopamine solution, the estimated CyanoCoating thickness is about 6 nm. The pDA layer thickness is within the range previously reported [24]. Regarding the PU, a thickness of 23 ± 4 nm including the pDA layer was obtained, which is not significantly different from the CyanoCoating result (Figure 1A). Even thicknesses obtained from different regions within the same sample showed that the coatings were homogeneous (data not shown). 

#### 2.2.2. Wettability

To determine the wettability of CyanoCoating and PU, captive bubble method (also named the inverted drop method) was applied. The results are presented in Figure 1B. CyanoCoating has a contact angle of 17 ± 2°, revealing a much higher hydrophilic nature (73% water contact angle reduction) than PU (65 ± 2°) and the pDA layer only (62 ± 12°) (Figure 1B). No significative differences were found between contact angles of PU and the pDA layer in accordance with what is reported by other authors [25,26].

#### 2.2.3. Surface Functional Groups

**Figure 2:**
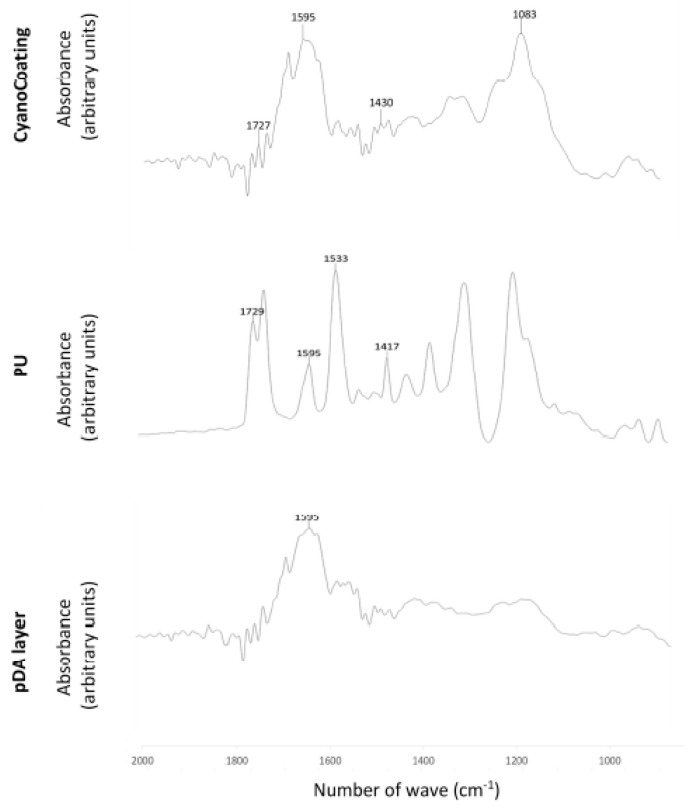
Fourier transform–infrared reflection absorption spectroscopy (FT–IRRAS) spectra of Au substrates coated with a polydopamine layer (pDA), a pDA layer plus polyurethane (PU), and a pDA layer plus the CyanoCoating. Typical peaks of each spectrum are highlighted.

To clarify which surface functional groups remain available after coating production, Fourier transform–infrared spectroscopy (FT–IR) analysis was performed. Figure 2 shows the FT–IR spectra obtained.

CyanoCoating exhibited polysaccharide-related peaks including, C-O-C vibration of the glucopyranose ring at 1083 cm^−1^, C-H bending typical of carbohydrates at 1430 cm^−1^, and C=O stretching related to uronic acids at 1727 cm^−1^ [18]. The observed intense peak at 1594 cm^−1^ is possibly related to the pDA layer underneath. Typical peaks from PU were observed, namely the absorption bands at 1533 cm^−1^ from N-H and C-N, at 1729 cm^−1^ from C=O associated to urethane groups, at 1417 cm^−1^ associated to the benzene ring, and 1595 cm^−1^ that can be also associated to the benzene ring or the pDA layer underneath [26]. In the pDA layer, the aforementioned peak at 1594 cm^−1^ assigned to H−N−C in-plane bending vibration was observed.

#### 2.2.4. Topography

The coatings topography was analysed by scanning electron microscopy (SEM). All coatings presented a smooth flat surface (Figure S1). 

#### 2.2.5. Surface Charge

The streaming potential method was employed to determine the ξ potential of the coatings. The ξ potential of CyanoCoating was −21 ± 6 mV. However, due to the low thickness of this film (about 6 nm) the ξ potential is probably influenced by the gold substrate (−38 ± 1 mV) and pDA layer (−23 ± 1 mV). The ξ potential obtained for PU was −17 ± 2 mV. Considering the chemical structure of Pellethane (the particular polyurethane used in this study) it was not expectable to have a negatively charged surface, suggesting again the influence of the underlying layers (pDA and Au). It is important to notice that the charge difference between CyanoCoating and PU is not statistically significant.

### 2.3. CyanoCoating Biological Performance

#### 2.3.1. Protein Adsorption

**Figure 3:**
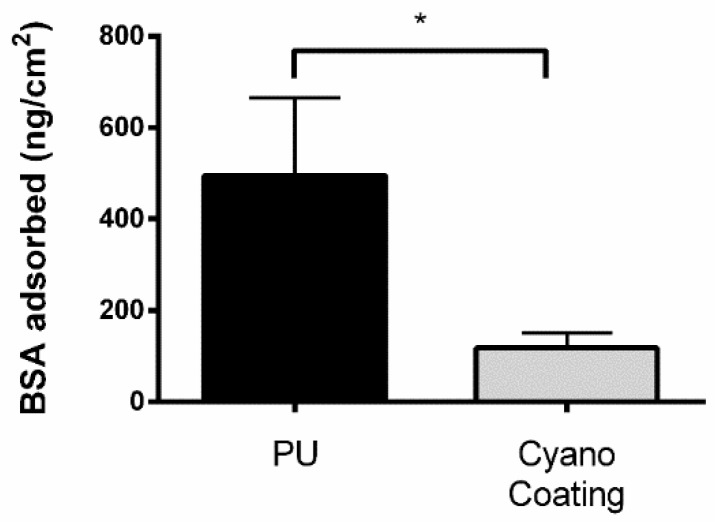
Adsorption of bovine serum albumin (BSA) onto polyurethane (PU), or CyanoCoating (Voigt modulation). Statistical significance between surfaces is indicated by * (*p* < 0.05, Unpaired t-test).

To evaluate non-fouling properties that may prevent protein adsorption, and consequently avoid the irreversible attachment of cells and/or bacteria [27,28,29], the CyanoCoating was challenged with model protein bovine serum albumin (BSA). The amount of BSA adsorbed to CyanoCoating was 117 ± 33 ng/cm^2^, much lower than the 495 ± 170 ng/cm^2^ observed for PU (Figure 3). Therefore, although not having strict non-fouling properties, CyanoCoating was able to significantly reduced protein adsorption (77%) compared to the reference material PU.

#### 2.3.2. Bacterial Adhesion

**Figure 4:**
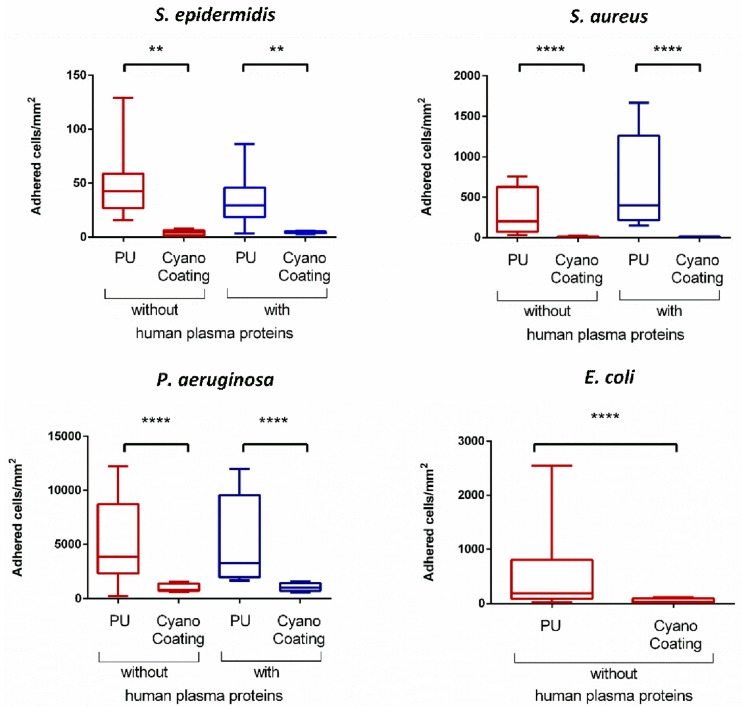
CyanoCoating anti-adhesive performance compared to medical grade polyurethane (PU), in the absence and presence of human plasma proteins. The coatings were tested against the four relevant etiological agents mentioned above each graph. The assay was performed according to ISO 22196. Statistical analysis was performed by Non-parametric Kruskal-Wallis analysis and statistic differences are indicated with ** (*p* < 0.01) and **** (*p* < 0.001).

The anti-adhesive performance of CyanoCoating was assessed against four relevant etiological bacterial agents in medical devices-associated infections: *Staphylococcus epidermidis*, *S. aureus* (Gram positive), *Pseudomonas aeruginosa* and *Escherichia coli* (Gram negative), according to ISO 22196:2007 (Plastics—Measurement of antibacterial activity on plastics surfaces). Overall, the results obtained showed that bacterial adhesion to CyanoCoating was greatly impaired compared to adhesion to PU (medical grade), ranging from 96% reduction for *S. aureus* to 80% reduction for *P. aeruginosa* (Figure 4). The presence of human plasma proteins, which has been reported as capable of promoting bacterial adhesion through the development of a protein layer with adhesive moieties [30], did not negatively impact the performance of CyanoCoating. With this coating, similar adhesion profiles were observed in the presence and absence of plasma proteins—ranging from 99% adhesion reduction for *S. aureus* to 80% reduction for *P. aeruginosa* (Figure 4). In contrast, the presence of plasma proteins promoted a 2-fold *S. aureus* adhesion increase to PU. For the *E. coli* anti-adhesive assays, plasma proteins were not used as this pathogen is mostly related to urological mucosa where plasma proteins are not an issue [31].

**Figure 5:**
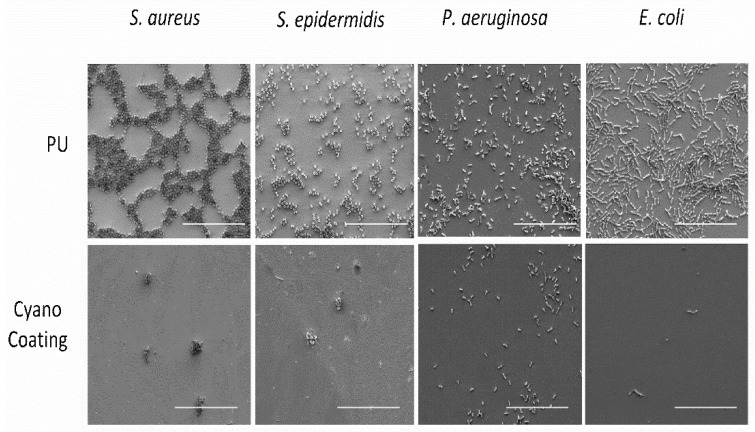
Scanning electron micrographs of polyurethane (PU) and CyanoCoating after 24 h incubation at 37 °C with four relevant etiological agents (scale bars—20 µm).

The results obtained for the bacterial adhesion assay were further supported by SEM analysis (Figure 5), in which a similar reduction trend can be observed.

**Figure 6:**
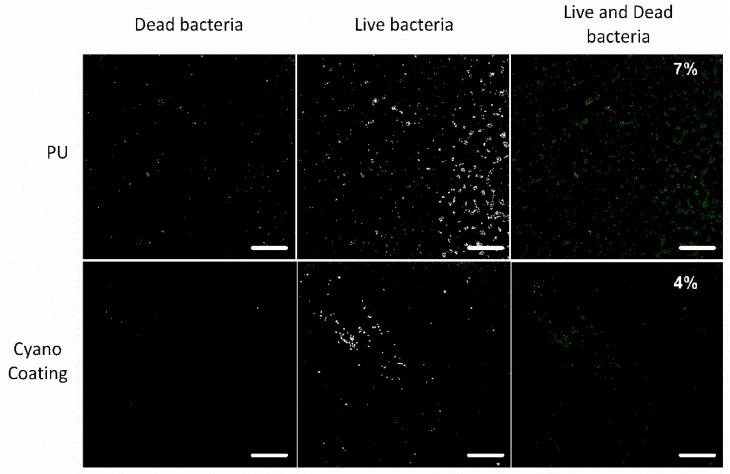
Micrographs of *Staphylococcus epidermidis* cells adhered to polyurethane (PU) and CyanoCoating after 24 h incubation at 37 °C and stained with Draq5 and propidium iodide (PI). In the right panel, the live bacteria are marked in green and the dead bacteria are marked in red. The percentage of dead bacterial cells is indicated (scale bars—60 μm).

In order to further clarify if the observed effect was derived from bactericidal properties of the CyanoCoating, a viability assay was performed. Representative images are depicted in Figure 6. As can be observed, the percentage of dead bacteria is very low (<7%) on both surfaces (CyanoCoating and PU) suggesting that none of them have a bactericidal effect.

#### 2.3.3. Platelet Adhesion and Activation

The effect of CyanoCoating on the adhesion and activation of platelets was followed by SEM, as the activation profile of adhered platelets is indicative of thrombus formation propensity [26,32]. Therefore, in this study, three different activation states were considered (non-activated, partial activated and fully activated) as depicted in Figure S2.

**Figure 7:**
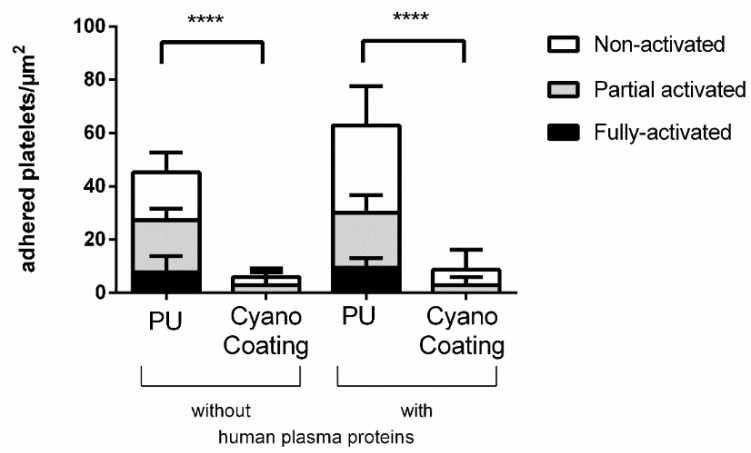
Number of adhered platelets to polyurethane (PU) and CyanoCoating per µm^2^ after 30 min of incubation at 37 °C in the presence or absence of human plasma proteins (n = 9). Statistical analysis was performed by non-parametric Kruskal–Wallis analysis and differences are indicated with **** (*p* < 0.001).

The quantitative distribution of platelets according to their activation state in CyanoCoating and PU is depicted in Figure 7.

The results obtained demonstrate that CyanoCoating reduces platelet adhesion in 87% compared to PU, even in the presence of human plasma proteins. In contrast, the number of adhered platelets to PU increased significantly (31%) in the presence of plasma proteins. Concerning the platelet activation profiles, CyanoCoating did not promote activation, since most adhered platelets were in low activation state (non-activated or partially activated, but none fully activated). In contrast, a high number of platelets adhered to PU exhibited partially or fully activated morphologies.

#### 2.3.4. Biocompatibility

**Figure 8:**
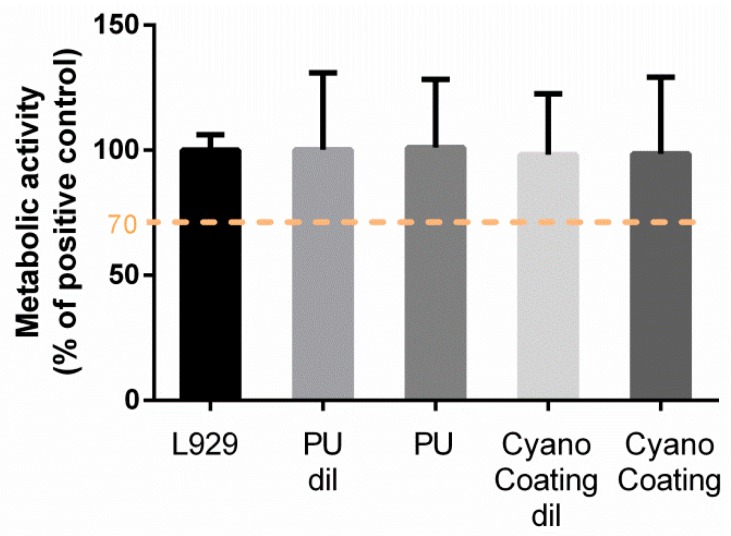
Metabolic activity of L929 mouse fibroblasts after 24 h incubation with polyurethane (PU) and CyanoCoating extracts diluted 1:1 (dil) or non-diluted, assessed using the MTT cytotoxicity test (n = 12). Statistical analysis was performed by a non-parametric Kruskal–Wallis analysis.

In order to evaluate the biocompatibility of CyanoCoating, L929 mouse fibroblasts were exposed to PU and CyanoCoating extracts, according to ISO 10993-5:2009 (Biological evaluation of medical devices-Tests for in vitro cytotoxicity). According to the ISO standard, metabolic activity below 70% is associated with cytotoxic potential. The metabolic activity of L929 cells in the presence of CyanoCoating extracts (non-diluted or diluted 1:1 in Modified Eagle’s medium (MEM)) was not statistically different from positive control, suggesting non-cytotoxicity against L929 fibroblast cells (Figure 8). 

## 3. Discussion

Medical device-associated infections remain one of the major problems in healthcare systems worldwide, so it becomes crucial to find novel effective solutions to address this pressing issue. In this context, coating the devices with natural polymers with anti-adhesive and/or antimicrobial properties is a current trend of research.

In this study, an extracellular polymer produced by a marine cyanobacterium, *Cyanothece* sp. CCY 0110 (hereafter *Cyanothece*), was used for the production of an effective anti-adhesive coating. *Cyanothece* was grown in conditions that promoted cell growth/released polysaccharides (RPS) production [18], and the amount of RPS produced was consistent with an efficient cyanobacterial-RPS producer [33,34]. This issue is of particular importance if, in the future, one wishes to produce/isolate this polymer at an industrial scale. The complex composition of the polymer was previously determined and consists of nine different monosaccharides, including galacturonic and glucuronic acids (7% of polymer dry weight), sulphate groups (11% of polymer dry weight), and peptides (4% of polymer dry weight) [19]. The production of a coating based on this extracellular polymer (CyanoCoating) was successful, as can be inferred by the results obtained with several surface characterization techniques (Figure 1, Figure 2 and Figure S1). CyanoCoating has a smooth topography, low thickness and high hydrophilic properties with mild negative charge. The low contact angle of CyanoCoating (Figure 1B) is consistent with values reported for other polysaccharides from marine sources such as alginic acid that shows a contact angle around 20° [35]. Regarding surface charge, no significant differences between CyanoCoating and reference PU surface were obtained. This result suggests that the amount of uronic acids and sulphate groups, or the orientation adopted by the RPS polymer chains after adhesion onto pDA-activated gold surfaces, did not contribute to promote a more negatively charged microenvironment than the observed on gold and pDA layer. CyanoCoating demonstrated low protein adsorption promotion compared to PU (77% less). Protein adsorption is described as being one of the first events after device implantation and, therefore, responsible for a conditioning film that modifies the physicochemical characteristics of material surfaces [27,28,29]. This affects the subsequent putative bacterial adhesion by non-specific (hydrogen bonding, hydrophobic, electrostatic, van der Waals [36]) or specific interactions by molecular recognition between bacteria adhesins and specific regions (peptide sequence Arginine, Glycine, Aspartate (RGDs)) of certain adsorbed proteins (like fibronectin and fibrinogen) [37]. CyanoCoating low protein adsorption value was not surprising due to its hydrophilic profile (water contact angle = 17 ± 2°). Surfaces with hydrophilic character resist the adsorption of proteins/cells due to the establishment of a hydration layer formed by well-structured water molecules linked to the surface by hydrogen bonds that works as a physical barrier [38]. As CyanoCoating is less prone to form a layer of adsorbed proteins, tight attachment of bacteria due to specific binding is less likely. Indeed, CyanoCoating significantly reduced the number of adhered bacteria (≤80%) even in the presence of plasma proteins. This decrease was observed for both Gram-positive and Gram-negative bacteria (Figure 5 and Figure 6), and we were able to demonstrate that the few bacterial cells that remained adhered to CyanoCoating were still viable (Figure 7), suggesting an anti-adhesive coating without antimicrobial capacity. Our results are in agreement with previous reports that demonstrated the anti-adhesive activity of natural polysaccharides from prokaryotic and eukaryotic sources [5,8,9,15,39]. This feature has already been explored to prevent *Helicobacter pylori* adhesion to gastric mucosa [5], and to develop coatings that prevent bacterial adhesion to orthopaedic medical devices [9,39]. Gadenne et al. [9] developed a coating based on ulvan (a sulphated polysaccharide extracted from green algae *Ulva rotundata* and *U. compressa*) that was applied to titanium, and was capable of reducing the adhesion of *P. aeruginosa* (80%) and *S. epidermidis* (96%) compared to non-modified substrate [9]. Shi et al. [39] also demonstrated that grafted oxidized dextran was able to reduce about 2-fold the adhesion of *S. epidermidis* and *S. aureus* to titanium. 

Here, CyanoCoating was further challenged with blood-contacting conditions. The results revealed that CyanoCoating anti-adhesive effect was also extensive to platelets. Indeed, past reports have pointed to the application of hydrophilic surfaces to prevent platelet adhesion and activation [26,40]. This observation was not dependent on the presence of plasma proteins, in contrast with the reference material (PU), where an increase in the number of adhered platelets was verified. This can be explained by the adsorption of proteins like fibrinogen, which is the third most abundant plasma protein in blood and plays a prominent role in developing surface-induced thrombosis [41,42]. Fibrinogen has in its composition platelet-binding sequences that can be exposed after its surface adsorption, leading to platelet binding and activation. Therefore, the improved results of CyanoCoating regarding platelet adhesion/activation in comparison to PU are very promising considering its application for blood-contacting medical devices, since the PU (Pellethane 2363 80 AE) selected as control is frequently used in cardiovascular applications, namely intravascular catheters [23].

Furthermore, the biocompatibility of CyanoCoating was established through the incubation of proliferating viable fibroblasts with the coating’s extracts. No toxicity was observed by measuring the metabolic activity of the cells. This non-cytotoxic behaviour is in accordance with the results described by Leite et al. [6], where no cytotoxicity in human dermal neonatal fibroblast cells was observed up to 0.12 mg/mL of *Cyanothece* sp. CCY 0110 polymer in solution.

## 4. Materials and Methods

### 4.1. Cyanobacterium Growth Conditions and Biopolymer Isolation

The unicellular cyanobacterium *Cyanothece* sp. CCY 0110 (kindly provided by Lucas Stal; Culture Collection of Yerseke, Yerseke, The Netherlands), now available at Culture Collection of Algae and Protozoa (CCAP 1435/2), was grown in 2 L bioreactors with ASNIII medium [43], at 25 °C, under a 16 h light (30 μE/m^2^/s)/8 h dark regimen, with orbital stirring (150 rpm) and aeration (1.2 L/min) [18], until an optical density (OD_730 nm_) of ≈2.5–3.5 was reached. 

For dry weight DW measurements, 5 mL of culture were collected and centrifuged for 10 min at 5000 rpm at room temperature (RT). The pellet was washed in type II water, another centrifugation step was performed, and the pellet was dried at 55 °C. 

For the isolation of the extracellular biopolymer the culture (≈1.5 L) was placed in dialysis membranes (12–14 kDa cut-off) and dialyzed against a minimum of 10 volumes of type II water for 48 h in continuous stirring. Cells were removed by high speed centrifugation (20,000 g, 15 min, 4 °C) and the supernatant was precipitated with two volumes of 99% ethanol (AGA) at 4 °C overnight. The biopolymer was collected with sterile metal forceps, dissolved in type II water, and precipitated once more [18]. The collected biopolymer was lyophilized (ECO D903, Labconco, Kansas City, MO, USA), grounded (mill A10 basic, IKA, Staufen, Germany) and stored in a desiccator until further steps.

### 4.2. CyanoCoating Development

#### 4.2.1. Substrate Preparation

Gold substrates (Au) production and cleaning were performed according to Martins et al. [44]. Briefly, chromium (5 nm) and gold (25 nm) layers were deposited by ion beam sputtering from chromium and gold targets (99.9% purity) on silicon wafers (AUREL, GmbH, Cham, Switzerland). Chromium was used to improve the adhesion of gold to silicon. Au substrates (1 × 1 cm) were cleaned with “piranha” solution (7 parts of sulfuric acid (95%, *v*/*v* (BDH Prolabo) 3 parts of hydrogen peroxide (Merck)), for 5 min (caution: this solution reacts violently with many organic materials and should be handled with suitable protective measures), thoroughly rinsed in a sonication bath (Bandelin Sonorex Digitec Bath 35 kHz, Berlin, Germany) for 3 min with ethanol and dried with a gentle stream of argon.

#### 4.2.2. Substrate Activation

Au substrates were immersed in freshly prepared dopamine solution (2-(3,4-Dihydroxyphenyl)ethylamine hydrochloride, Sigma-Aldrich, Darmstadt, Germany) (2 mg/mL in 10 mM Tris-HCl pH 8.5) and incubated for 2 h with orbital shaking (70 rpm) in the darkness to allow polymerization of dopamine into a polydopamine (pDA) layer on top of the Au plate (Figure S3A). Subsequently, samples were rinsed in a sonication bath (Bandelin Sonorex Digitec Bath 35 kHz, Berlin, Germany) for 1 min, with Tris-HCl 10 mM pH 8.5 buffer (twice) and with type I water (once). Au substrates coated with pDA layer were dried with argon and stored no more than 2 h before use.

#### 4.2.3. Polyurethane (PU) Coating Production

Commercially available medical grade polyurethane (PU), Pellethane 2363 80 AE (Velox), was obtained in pellets form. The pellets were sonicated for 15 min (Sonorex Digitec Bath 35 kHz, Bandelin, Berlin, Germany) twice with hexane (Merck, Darmstadt, Germany) and once with ethanol 99% (Merck). This process was repeated twice to remove any trace of silicone from of the extrusion process of the PU pellets. Afterwards, the pellets were dried in a vacuum oven (Trade Raypa, Barcelona, Spain), at RT overnight. A PU solution 0.1% (*w*/*v*) was prepared in tetrahydrofuran (Merck). PU coated samples were prepared using a spin-coater (model WS-650-23, Laurell Technologies Corp., North Wales, PA, USA), at 9000 rpm for 1 min, to apply the PU solution on top of pDA-coated Au substrate (Figure S3B).

#### 4.2.4. CyanoCoating Production

A biopolymer solution at 0.5% (*w*/*v*) was obtained by hydrating the lyophilized biopolymer in type II water for 24 h at RT, and then autoclaved at 110 °C for 30 min. After cooling, the biopolymer solution was spin-coated (model WS-650-23, Laurell Technologies Corporation, North Wales, PA, USA) at 9000 rpm for 1 min on top of pDA-coated Au substrate (Figure S3C).

### 4.3. Surface Characterization

Before surface characterization, CyanoCoating and PU were dried in a vacuum oven (Trade Raypa, Barcelona, Spain), at RT for at least 1 h.

#### 4.3.1. Ellipsometry

Ellipsometry measurements were performed using an imaging ellipsometer (EP3, Nanofilm Surface Analysis, Accurion, Goettingen, Germany). The ellipsometer operated in a polarizer-compensator-sample-analyser (PCSA) mode (null ellipsometry). The light source was a solid-state laser with a wavelength of 532 nm. The gold substrate refractive index (n = 0.681) and extinction coefficient (k = 2.478) were determined by using a delta and psi spectrum with an angle variation between 60 and 81°, and a brewster angle of 70°. These measurements were performed in one zone. The thickness of the CyanoCoating and PU were determined considering n = 0.638 and k = 2. Results are the average of two measurements of three replicates of three independent assays.

#### 4.3.2. Water Contact Angle (WCA)

Water contact angle measurements were performed using the captive bubble method with a goniometer model OCA 15, equipped with a video CCD-camera and SCA 20 software (Data Physics, Filderstadt, Germany). CyanoCoating and PU were tape glued to a microscope slide and placed in a quartz chamber filled with type I water. Subsequently, 10 μL bubbles of room air were release from a J-shaped needle placed underneath the sample surface at a dose rate of 2 µL/s. Bubble profiles were fitted using different mathematical functions. Results are the average of two measurements of three replicates of three independent assays.

#### 4.3.3. Fourier Transform–Infrared Reflection Absorption Spectroscopy (FT–IRRAS)

Measurements were performed on a Fourier transform infrared spectrophotometer (model 2000, Perkin Elmer, Waltham, MA, USA) coupled to a VeeMax II Accessory (PIKE Technologies, Madison, WI, USA) and a liquid-nitrogen-cooled mercury cadmium telluride (MCT) detector. In order to ensure that there was no water vapor adsorption, dry nitrogen was purged into the instrument for 5 min before and during the measurement of each sample. For each substrate, a similar gold surface was used as a background. Incident light was p-polarized and spectra were collected using the 80° grazing angle reflection mode. For each sample, 100 scans were collected with 4 cm^−1^ resolution.

#### 4.3.4. Scanning Electron Microscopy with Energy-Dispersive Spectroscopy (SEM/EDS)

The SEM/energy-dispersive spectroscopy (EDS) analysis was performed using a high-resolution (Schottky) environmental scanning electron microscope with X-ray microanalysis and electron backscattered diffraction analysis (Quanta 400 FEG ESEM/EDAX Genesis X4M, FEI Company, Hillsboro, OR, USA), at CEMUP (Centro de Materiais da Universidade do Porto). Micrographs of the surfaces were taken using an electron beam intensity of 5 kV (accelerating voltage) and a magnification of 5000×. To increase the surface conductivity, samples were previously sputtered with gold/palladium (Au/Pd) for 60 s and 15 mA current using the SPI module sputter coater equipment (Structure Probe Inc., West Chester, PA, USA).

#### 4.3.5. Electrokinetic Analyzer

The zeta potential (ξ) of the samples was determined from streaming potential measurements with a commercial electrokinetic analyser (EKA) (Anton Paar GmbH, Graz, Austria) using the so-called “Stamp Cell”, designed for small rectangular samples, as described elsewhere [45]. The Stamp Cell is composed of two poly(methyl methacrylate) (PMMA) sample holders with a cross-section of 1 × 2 cm^2^. A defined gap between the sample surfaces is achieved by means of a micrometre screw. The electrolyte is circulated through this gap, thereby creating a differential pressure to shear off the diffuse part of the electrochemical double layer at the sample/electrolyte interface. The streaming potential was measured by Ag/AgCl electrodes, which are installed at both ends of the streaming channel. The sample is fixed on the respective PMMA piston with a double-sided adhesive tape for measurement. The electrolyte used was KCl (VWR), 1 mM and pH 7, and experiments were performed at RT. The conductivity of the electrolyte solution was measured during the experiments. The streaming potential was measured while applying an electrolyte flow with alternative direction and pressure ramps from 0 to 400 mbar. For each measurement, 12 pressure ramps were performed (six in each flow direction). The ξ potential was calculated according to the Fairbrother–Mastin method, which is included in the software package (VisioLab, version 1.03.3507, Toulouse, France) of the equipment.

### 4.4. CyanoCoating Biological Performance Evaluation

#### 4.4.1. Protein Adsorption Studies: Quartz Crystal Microbalance with Dissipation (QCM–D)

Gold-coated quartz crystal microbalance with dissipation (QCM–D) sensors (fundamental frequency of 5 Mhz; Biolin Scientific, Gothenburg, Sweden) were cleaned as previously described [46]. Briefly, the sensors cleaning procedure consisted in a 10 min oxidation in an ultraviolet (UV) oven, followed by immersion in a “piranha” solution (see Section 4.2.1). Afterwards, sensors were rinsed and sonicated for 3 min in type II water and dried with a gentle argon stream. After cleaning, the sensors were used as substrate for coatings production as explained previously (Section 4.2.2). A QCM–D system (Q-Sense E4 instrument, Biolin Scientific, Gothenburg, Sweden) was used to monitor in real time the frequency (∆f) and dissipation (∆D) shifts on the sensors related to protein adsorption. Samples were pre-incubated with phosphate buffered saline solution (PBS) in static conditions for 15 min to establish a baseline. Afterwards, bovine serum albumin (BSA) (Sigma-Aldrich) at 4000 μg/mL in PBS, was individually injected in the QCM–D modules in continuum, at a rate of 25 μL/min (37 °C). The flow was maintained until saturation was reached (≈40 min). PBS solution was also used to remove unattached protein molecules after saturation (≈15 min). Data was modelled using the Voigt model, since it takes into account the viscoelastic contributions of the hydrated layer. Data from the 3rd to the 11th harmonics were collected and used in the analysis. The density and viscosity of the protein solutions were established at 1.35 g/cm^3^ and 0.0014 kg/ms (values commonly used for low concentrated protein solutions [47,48]). Results are presented in mass per area (ng/cm^2^) and represent the average of three independent assays with three replicates per sample.

#### 4.4.2. Bacterial Assays

##### 4.4.2.1. Bacterial Strains, Media and Growth Conditions

*Staphylococcus epidermidis* (ATCC 35984), *S. aureus* (ATCC 33591), *Pseudomonas aeruginosa* (ATCC 27853) and *Escherichia coli* (ATCC 25922) were obtained from the American Type Culture Collection (Manassas, VA, USA). Bacteria were grown on tryptic soya agar (TSA) (Merck) and tryptic soya broth (TSB) (Merck). Initial bacterial inoculum was prepared in TSB, after measuring OD_600 nm_ and subsequently confirmed by retrospective count of colony forming units (CFUs). 

##### 4.4.2.2. Bacterial Adhesion Assays

Bacterial adhesion assays were performed according to ISO 22196:2007 (Plastics—Measurement of antibacterial activity on plastics surfaces). CyanoCoating and PU were incubated twice with ethanol 70% (Merck, Darmstadt, Germany) and twice with filtered type II water (0.22 μm syringe filter) for 15 min. Samples were dried with argon stream in a flow hood and then transferred to a 24-well plate. A 5 µL inoculum drop (1.8 × 10^6^ CFUs/mL with or without 1% (*v*/*v*) human plasma proteins (human plasma was obtained through an automated blood processing system, Reveos^®^ Terumo BCT, Tokyo, Japan and was kindly provided by Hospital de São João, Porto, Portugal) was placed on top of the samples and then covered with a previously sterilized polypropylene (PP) coverslip (Ø 9 mm). Samples were incubated for 24 h at 37 °C in moisturized condition. After 24 h, samples were rinsed with PBS three times. Adhered bacteria were fixed with paraformaldehyde 4% (*v*/*v*) in PBS, for 30 min at RT. After rinsing with PBS three times, samples were stained with 4′,6-diamidino-2-phenylindole (DAPI) (0.1 μg/mL, Sigma-Aldrich, St. Louis, MO, USA) for 30 min at RT, protected from light. Afterwards, samples were rinsed with PBS and transferred to an uncoated 24-well μ-plate (#82406, IBIDI, Martinsried, Germany) with the surface facing the bottom. Results represent average of three independent assays, with three replicates per sample.

##### 4.4.2.3. Bacterial Viability Assay

To assess the viability of adhered bacteria to the coatings, a bacterial adhesion assay was performed as described in the Section 4.4.2.2. After the 24 h incubation period, samples were rinsed twice with sterile PBS and stained with Draq5 (5 μM, BioStatus, Shepshed, UK) and propidium iodide (PI) (1.25 μg/mL, Molecular Probes, Eugene, OR, USA), at RT in darkness. Draq5 stains all bacterial cells while PI can only penetrate damaged cells membranes. Afterwards, samples were rinsed with PBS and transferred to an uncoated 24-well μ-plate (#82406, IBIDI, Martinsried, Germany) with the surface facing downwards. Three replicates of each sample were analysed.

##### 4.4.2.4. Image Acquisition and Analysis

Samples from bacterial adhesion assay were observed using high-content screening microscope (IN Cell Analyzer 2000, GE Healthcare, Chicago, IL, USA) with a Nikon 20×/0.95 NA Plan Apo objective (binning 1 × 1), using a CCD Camera (CoolSNAP K4, Teledyne Technologies, Thousand Oaks, CA, USA). Image field of view (FOV) x-y for this objective is 0.8 × 0.8 cm. 9 FOV per sample were acquired spanning an area of 5.76 cm^2^. The excitation and emission filters used were DAPI (excitation: 365 nm; emission: 420 nm). On-the-fly deconvolution was performed. The number of adherent bacteria were quantified using the ImageJ software, and values were converted to bacteria per mm^2^.

Images from the bacterial viability assay were acquired using the same high-content screening microscope but with a Nikon 40×/0.95 NA Plan Apo objective (binning 1 × 1), using a CCD Camera (CoolSNAP K4, Teledyne Technologies, Thousand Oaks, CA, USA). Image field of view (FOV) x-y for this objective is 0.04 × 0.04 cm. 9 FOV per sample were acquired spanning an area of 1.44 mm^2^; each comprising a z-section of 16.5 µm (z-slice = 11, z-step = 1.5 µm). The excitation and emission filters used to detect PI and Draq5 were Cy3 (605 nm) and Cy5 (705 nm), respectively. Post-acquisition image deconvolution was performed in the IN Cell Analyzer 2000 5.2-14311 (GE Healthcare, Chicago, IL, USA) software.

Original images z-sections were projected on one plane using the IN Cell Investigator Developer Toolbox v. 1.9.2 (GE Healthcare, Chicago, IL, USA). For bacteria identification, a software package for image segmentation running a machine-learning algorithm—ilastik was used [49]. Customized algorithms were generated for each wavelength and individual signal probability images were generated for each projected image. Probabilities images were fed into CellProfiler version 3.1.5 [50] and number of viable and dead bacteria was quantified.

##### 4.4.2.5. Scanning Electron Microscopy (SEM) analysis of Adhered Bacteria

Bacterial adhesion assays were performed according to 4.4.2.2. At the end of the incubation period, surfaces were rinsed with PBS and the adhered bacteria were fixed with 1.5% (*v*/*v*) glutaraldehyde (Merck) in 0.14 M sodium cacodylate buffer (Merck) for 30 min at RT. Then, bacteria-adhered surfaces were dehydrated in a growing ethanol/water gradient: 50, 60, 70, 80, 90 and 99% (*v*/*v*), for 10 min each. Finally, the bacteria-adhered surfaces were dried by critical point drying, using a series of temperature variations from 4 °C to 33−38 °C until a maximum pressure of 1000−1400 psi was reached. The films conductivity was enhanced by sputtering with Au/Pd for 60 s and 15 mA current using the SPI Module Sputter Coater equipment (Structure Probe, Inc, West Chester, PA, USA). The SEM analysis was performed using a high-resolution SEM with X-ray microanalysis: JSM 6301F (Jeol, Peabody, MA, USA) at 5 kV and magnification of 5000×, at CEMUP.

#### 4.4.3. Platelet Adhesion and Activation Assay

CyanoCoating and PU thrombogenicity potential was assessed according to ISO 10993-4:2009 (Biological evaluation of medical devices - Selection of tests for interactions with blood). Platelets from an intermediary platelet unit (IPU), provided by Hospital de São João (Porto, Portugal) were used. Samples were sterilized as described in Section 4.4.2.2. After sterilization, samples were immersed in 1% (*v*/*v*) human plasma in PBS, for 30 min at 37 °C, and rinsed three times with PBS. Simultaneously, 24-well tissue culture polystyrene plates (TCPS, Sarstedt, Nümbrecht, Germany) were incubated at 37 °C with 1% (*w*/*v*) BSA in PBS for 1 h, to reduce platelet activation in response to the oxidized TCPS. Protein-adsorbed surfaces were transferred to the BSA-treated plates, previously washed five times with PBS, and incubated with IPU at 3 × 10^8^ platelets/mL in PBS for 30 min at 37 °C and 70 rpm. Surfaces were rinsed with PBS and processed for SEM analysis as explained in 4.4.2.5. The SEM analysis was performed using a high-resolution SEM with X-ray microanalysis: JSM 6301F (Jeol, Peabody, MA, USA) at 5 kV, at CEMUP. Five micrographs of each surface were taken at 2000× magnification (n = 9). The number of platelets and different activation stages per surface condition were quantified using the ImageJ software and reported as platelets/mm^2^.

#### 4.4.4. Biocompatibility Assay

The biocompatibility of CyanoCoating and PU extracts was evaluated according to ISO 10993-5:2009 (Biological evaluation of medical devices-Tests for in vitro cytotoxicity), using L929 mouse fibroblasts cell line (ATCC). The L929 cells were seeded in modified Eagle’s medium (MEM, Gibco) (supplemented with 10% (*v*/*v*) Fetal Bovine Serum (Gibco) and 1% (*v*/*v*) penicillin/streptomycin (Biowest)) at 1 × 10^5^ cells/mL and maintained at 37 °C in humified atmosphere of 5% CO_2_ for 24 h. 

CyanoCoating and PU extracts were prepared according to ISO 10993-12:2004 (Biological evaluation of medical devices—Sample preparation and reference materials). Samples were incubated in MEM media (without supplementation) at 37 °C in a humified atmosphere of 5% CO_2_ for 24 h. Extracts were used non-diluted and diluted 1:1 (*v*/*v*) in MEM. Dimethylsulfoxide (DMSO) diluted 1:1 (*v*/*v*) in MEM was used as control. After 24 h, L929 cell growth MEM medium was replaced by extracts, and re-incubated for another 24 h. Then, supernatants were removed and 50 µL of MTT (1 mg/mL 3-(4,5-dimethylthiazol-2-yl)-2,5-diphenyltetrazolium bromide freshly prepared in MEM incomplete (non-supplemented with Fetal Bovine Serum and penicillin/streptomycin)) was added to each well and incubated for 2 h at 37 °C. MTT was metabolically reduced by viable cells to a blue-violet insoluble formazan. Afterwards, MTT was removed and DMSO was added to solubilize formazan. Finally, photometric measurements were performed at 570 nm in a microplate reader using BioTek Synergy Mx (Molecular Devices, San Jose, CA, USA). Four replicates for each condition were used.

## 5. Conclusions

Cyanobacteria, in particular marine strains, represent potential sources of polymers with biomedical applications. The extracellular polymer produced by *Cyanothece* sp. CCY 0110 was successfully applied to develop a promising broad-spectrum anti-adhesive coating, whose blood-compatibility and biocompatibility may allow its application to a wide range of medical devices. Prevention of bacterial adhesion to surfaces through anti-adhesive coatings is one of the simplest and potentially most cost-effective ways to avoid biofilm formation and subsequent infection. The exploitation of coatings based on marine polymers may constitute a step further in the current struggle to find alternative infection-fighting measures without the administration of antibiotics.

## Supplementary Materials

The following are available online at https://www.mdpi.com/1660-3397/17/4/243/s1: Figure S1: Scanning electron micrographs of the Au substrates coated with a polydopamine (pDA layer), a pDA layer plus polyurethane (PU), and a pDA layer plus the CyanoCoating. The residual debris on the CyanoCoating were identified as calcium and magnesium salt crystals using energy dispersive X-ray spectroscopy (EDS), and are most probably remains from the Cyanothece’s culture medium (scale bars: 20 μm). Figure S2: Representative SEM micrographs of platelets adhered to polyurethane (PU) and categorized by activation state: (A) Non-activated, (B) Partially activated and (C) Fully activated (scale bars: A—4 μm, B—7 μm, C—6 μm). Figure S3: Schematic representation of samples characterized in this study: gold substrates coated with a polydopamine (pDA) layer, a pDA layer plus polyurethane and a pDA layer plus CyanoCoating.
