# Supplementary material for: Broad-Spectrum Anti-Adhesive Coating Based on an Extracellular Polymer from a Marine Cyanobacterium
Source: Mar Drugs. 2019 Apr 24;17(4):243. doi: 10.3390/md17040243 (PMC6520837; doi:10.3390/md17040243)
Supplement: Supplementary file 1 [file marinedrugs-17-00243-s001.pdf]

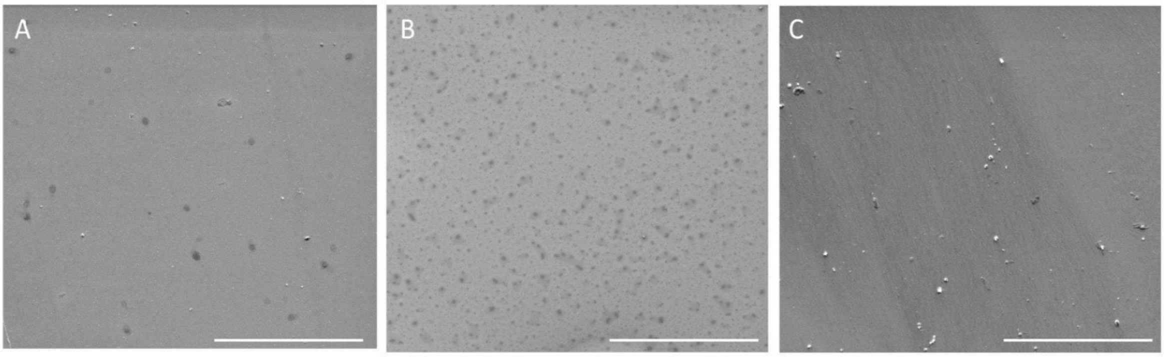

Figure S1: Scanning electron micrographs of the Au substrates coated with a polydopamine (pDA layer), a pDA layer plus polyurethane (PU), and a pDA layer plus the CyanoCoating. The residual debris on the CyanoCoating were identified as calcium and magnesium salt crystals using energy dispersive X-ray spectroscopy (EDS), and are most probably remains from the *Cyanothece*'s culture medium (scale bars - 20 µm).

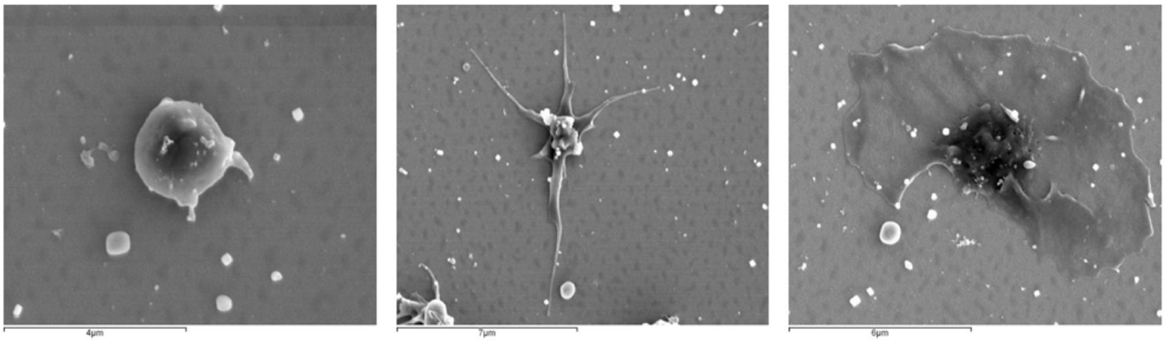

Non-activated Partial activated Fully activated

Figure S2: Representative SEM micrographs of platelets adhered to polyurethane (PU) and categorized by activation state: Non-activated, Partially activated and Fully activated (scale bars: A - 4 µm, B - 7 µm, C - 6 µm).

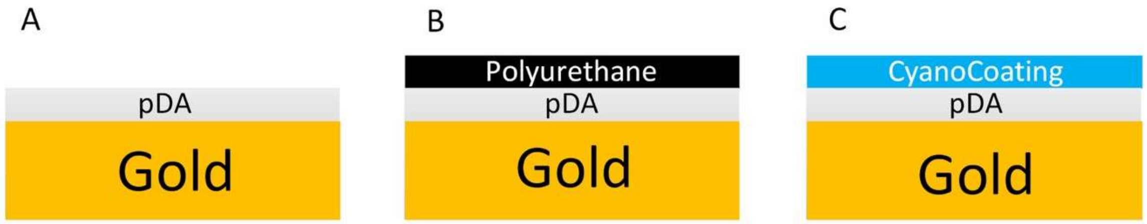

Figure S3: Schematic representation of samples characterized in this study: gold substrates coated with (**A**) a polydopamine (pDA) layer, (**B**) a pDA layer plus polyurethane and (**C**) a pDA layer plus CyanoCoating.
